# Reforming China’s Rare Disease Security System: Risk Management Perspectives and a Dedicated Insurance Innovation

**DOI:** 10.3390/healthcare13172178

**Published:** 2025-08-31

**Authors:** Yumeng Zhang, Minghao Yang, Qiang Su, Yuanhao Sui, Lihua Sun

**Affiliations:** Department of Pharmacy Administration, School of Business Administration, Shenyang Pharmaceutical University, 103 Wenhua Road, Shenyang 110016, China; ymzhang202204@163.com (Y.Z.); ymh9077@163.com (M.Y.); 13540168209@163.com (Q.S.); 15564199762@163.com (Y.S.)

**Keywords:** rare diseases, risk management theory, health capital model, dedicated insurance scheme, multi-tiered medical security system, institutional reform, health policy

## Abstract

**Objectives**: Patients with rare diseases in China face extremely high medical expenses. The current coverage framework remains inadequate in terms of coverage depth and proactive risk control, underscoring an urgent need for institutional reform. **Methods**: This study employs a policy content analysis approach to review the current landscape of rare disease protection in China. Drawing on risk management theory and the health capital model, it constructs an analytical framework to examine potential institutional reforms through the lens of risk response pathways and the efficiency of health investment. **Results**: The findings reveal that basic medical insurance (BMI) provides limited financial protection for patients with rare diseases. Among China’s 31 provincial-level administrative centers, 24 have set general outpatient reimbursement ceilings under the urban and rural resident basic medical insurance (URRBMI) at 1000 RMB or less. In comparison, 24 cities have set outpatient reimbursement limits under the urban employee basic medical insurance (UEBMI) at 6000 RMB or less. The security system relies predominantly on the BMI, while supplementary mechanisms have failed to provide effective support or continuity in coverage. Current policies are generally reactive, with coverage typically triggered only after a confirmed diagnosis and often lacking early intervention or preventive strategies. **Conclusions**: China’s rare disease security system urgently requires structural improvements in coverage depth and proactive risk management. The proposed Dedicated Insurance Scheme for Rare Diseases (DISRD) presents a feasible and sustainable model for China’s multi-tiered system of securing rare diseases. It provides valuable institutional insights for other countries and regions seeking to build public health systems with proactive risk control capabilities.

## 1. Introduction

Rare diseases refer to a group of conditions that are characterized by extremely low prevalence, complex pathogenesis, diagnostic challenges, and often prohibitively high treatment costs [1,2]. In recent years, rare diseases have emerged as a focal issue in global public health and healthcare policy. According to the World Health Organization (WHO), a rare disease is defined as one that affects between 0.065% and 0.1% of the general population [3]. Despite the low incidence of individual diseases, the cumulative burden is substantial due to the sheer number of conditions that exist. According to data from the Orphanet database [4], over 7000 rare diseases have been identified globally, affecting an estimated 260 to 450 million individuals worldwide. Approximately 69.9% of these diseases have an exclusively pediatric onset, defined as initial manifestation occurring between birth and 18 years of age [4], and around 30% of affected children die before the age of five. In China, more than 20 million individuals live with rare diseases, with over 200,000 new cases diagnosed each year [5].

Given the small patient populations and limited commercial interest, rare diseases have historically received insufficient attention in pharmaceutical research and development (R&D) and market access. This lack of focus has led to few incentives for innovation, high drug prices, and underdeveloped reimbursement systems, posing a continuing policy challenge. Globally, only about 5% of rare diseases have effective treatments available [6]. Even when such drugs reach the market, their pricing is often remarkably high, as it is intended to recover substantial R&D investments, thereby imposing an enormous financial burden on patients [7].

In recent years, the Chinese government has increasingly prioritized the protection of patients with rare diseases, achieving initial progress through the expansion of the National Reimbursement Drug List (NRDL), the institutionalization of drug price negotiations, and the issuance of standardized clinical guidelines for the diagnosis and treatment of rare diseases. Some high-cost rare disease medications have been included in the NRDL, alleviating part of the financial pressure on patients. However, a considerable number of expensive, rare disease medications remain outside the reimbursement system, requiring patients to bear the full cost of treatment and resulting in substantial economic hardship. Table 1 highlights a selection of rare disease drugs that have not yet been included in the NRDL. The annual treatment costs of these drugs commonly range from several hundred thousand to over one million RMB, illustrating the formidable financial challenge faced by patients.

According to data from the China Organization for Rare Disorders (CORD), based on a survey of 5810 patients, the average annual out-of-pocket (OOP) expenditure for rare disease treatment exceeds RMB 40,000. Moreover, over 80% of respondents reported that treatment costs accounted for more than 80% of their total household income—far surpassing the 40% threshold defined by the World Health Organization (WHO) for catastrophic health expenditure [8,9]. Given the distinct characteristics of rare diseases—namely, high treatment costs, low prevalence, and a high risk of disability—there is an urgent need to establish a more forward-looking, structurally adaptive, and fiscally sustainable multi-tiered protection framework tailored to their unique risk profile.

Globally, an increasing number of countries have implemented supplementary mechanisms to protect individuals with rare diseases, such as Belgium’s Special Solidarity Fund [10], Australia’s Life Saving Drugs Program [11], Japan’s Medical Care Program for Specific Diseases [12], Taiwan’s tobacco-tax-funded Rare Disease Prevention and Control Fund [13], and commercial orphan drug coverage in the United States [14,15]. These models are typically based on centralized fiscal authority, statutory entitlement frameworks, or mature private insurance markets. In contrast, China’s decentralized health financing system, characterized by central guidance and local implementation, lacks a unified budget authority and statutory foundation for such top-down mechanisms. As such, these international models are not directly transplantable to the Chinese context.

Against this backdrop, this study advocates for the establishment of a rare disease-specific insurance framework aligned with China’s governance structure and fiscal capacity. Grounded in risk management theory and the health capital model, this framework is intended to complement, rather than replace, the existing medical insurance system, offering a more forward-looking and sustainable pathway for rare disease protection.

## 2. Materials and Methods

### 2.1. Research Design and Data Sources

This study adopts policy content analysis to systematically examine national policy documents related to healthcare security for rare diseases in China, aiming to identify key institutional features and policy gaps in coverage design and implementation.

Policy documents were collected from the official websites of the General Office of the State Council, the National Health Commission (NHC), the National Healthcare Security Administration (NHSA), the Ministry of Finance (MOF), and the Ministry of Civil Affairs (MCA), covering the period from 2012 to 2025. The search utilized keywords such as “rare disease,” “medical insurance,” “outpatient reimbursement,” and “orphan drugs.”

Inclusion criteria:

(1) Documents explicitly addressing rare disease-related healthcare coverage;
(2) Formally issued normative documents (e.g., notices, measures, guidance opinions, or implementation plans).

Exclusion criteria:

(1) Exact duplicates (e.g., the same policy published on multiple platforms, retaining only one copy);
(2) Non-complete official documents (e.g., news summaries, brief notices, or excerpts without the full text);
(3) Documents retrieved through a keyword search but containing no substantive content related to rare disease healthcare coverage.

For the national-level analysis, the 15 national-level documents were analyzed thematically, leading to the identification of four key thematic categories: (1) payment mechanisms (e.g., outpatient caps, reimbursement ratios, reimbursement standards); (2) inclusion of diseases and drugs (e.g., directory access criteria); (3) intervention timing (e.g., pre- vs. post-diagnosis entry points); and (4) institutional structure (e.g., integration with basic medical insurance or supplementary programs). Two researchers independently reviewed and manually coded each document line-by-line across the four dimensions. The coders read each line and classified relevant information according to the established thematic categories. Any text not applicable to the research questions (e.g., background information, introductory sections, or unrelated policy discussions) was excluded from the coding process, ensuring that only directly relevant content was categorized. In the initial independent coding stage, the coders achieved over 90% agreement. Discrepancies were resolved through discussion until a complete consensus was reached.

In addition to the national-level policy analysis, provincial-level data were also extracted to understand the actual status of rare disease healthcare coverage across different provinces. For the provincial-level analysis, the 62 provincial-level documents were used to extract quantitative information on outpatient reimbursement ceilings as of May 2025 for the 31 provincial-level administrative centers in mainland China, including four centrally administered municipalities, 22 provincial capitals, and five autonomous regional capitals. These regions were selected for their central role in local healthcare policymaking, data availability, and concentration of medical resources, making them representative of the highest standards within their respective provinces. Data extraction was conducted independently by two researchers using a cross-validation procedure, with discrepancies verified against authoritative sources. Metadata, including policy titles, official access links, and the specific reimbursement ceiling amounts for outpatient services across provinces, is provided in Supplementary Table S1.

### 2.2. Analytical Framework: Risk Management and Health Capital

#### 2.2.1. Risk Management Theory

Initially developed by Williams and Hans (1964) [16] (pp. 168–207), risk management theory emphasizes minimizing expected loss at the lowest cost through five core strategies: risk avoidance, loss control, risk dispersion, risk retention, and risk transfer.

Below, we briefly explain each strategy:

(1) Risk avoidance refers to eliminating the source of risk, preventing the adverse event from occurring.
(2) Loss control involves reducing the frequency or severity of potential losses through preventive or mitigating actions.
(3) Risk dispersion spreads risk across multiple individuals, systems, or processes to reduce concentration and vulnerability.
(4) Risk retention means that individuals, organizations, or systems bear the losses themselves in the absence of external risk-sharing mechanisms.
(5) Risk transfer reallocates the financial consequences of risk to a third party, typically through insurance or contractual arrangements.

In simple terms, this framework enables policymakers to design smarter and more proactive interventions by determining how to prevent risks, mitigate harm, and allocate costs through institutional mechanisms.

In the context of rare diseases—which are typically low in prevalence but high in financial and social burden—this framework provides a structured approach to selecting appropriate policy tools. It supports a shift from passive compensation after diagnosis to early-stage interventions that aim to slow down deterioration and reduce long-term system costs.

#### 2.2.2. Health Capital Model

Grossman’s health capital model (1972) [17] conceptualizes health as a form of human capital that depreciates over time. According to this model, individuals are born with an initial stock of health capital, which naturally declines with age and accelerates under poor health conditions. Notably, the model also suggests that the marginal efficiency of health investment—that is, the health gain per unit of input—diminishes with both increasing age and worsening health status. In other words, as individuals age or experience declining health, the returns on health investments become less effective, meaning that delayed care often results in higher costs and poorer outcomes.

Put simply, this model highlights why intervention early is not only medically practical but also economically wise—delaying care leads to higher costs and lower returns.

In the context of rare diseases—many of which have childhood onset, high diagnostic delay, low initial health capital, and accelerated clinical deterioration—early intervention can significantly enhance the cost-effectiveness and clinical efficacy of medical investment. The model thus provides strong theoretical justification for treating early-stage intervention as a high-return health investment window in the design of rare disease policy.

### 2.3. Integration of Theoretical and Methodological Approaches

This study adopts an integrated analytical framework that combines risk management theory with Grossman’s health capital model. Together, the two form a dual theoretical perspective for identifying the shortcomings of current rare disease coverage and designing targeted reform strategies. Although each theoretical framework provides different insights, they also overlap and merge in practice. A typical example of this convergence is early intervention. In risk management theory, early intervention embodies the core function of loss control—reducing the frequency or severity of potential losses through preventive measures. Simultaneously, it aligns with the logic of health capital accumulation: investing in health at the earliest stages of life yields greater returns in terms of clinical outcomes, quality of life, and long-term cost-effectiveness.

**Figure 1:**
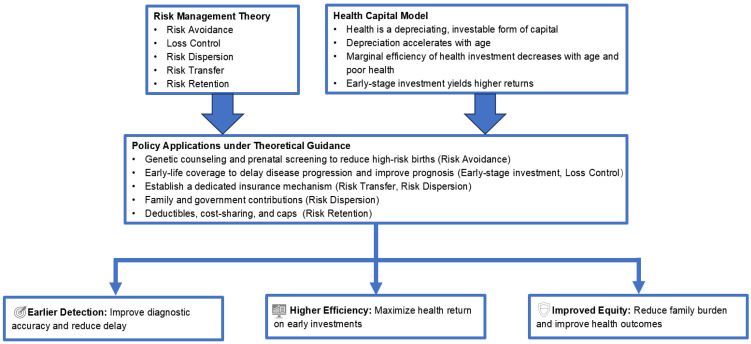
Integrated analytical framework based on risk management theory and the health capital model.

By anchoring the policy content analysis in this dual theoretical framework, this study enhances the rigor and practical Significance of the analysis. It enables us to interpret rare disease policies not only as a response to institutional fragmentation or fiscal burden, but also as a strategic decision to manage risks and maximize long-term health capital. As shown in Figure 1, this dual perspective informs policy design in the rare disease context, supporting three overarching goals: earlier detection, greater efficiency, and enhanced equity.

## 3. Results

Based on the collected policy documents, this section conducts a systematic analysis of both central-level directives and local implementation practices across several institutional dimensions, including payment mechanisms, inclusion criteria, coverage structures, and the timing of intervention. The titles, publication dates, thematic categories, and relevant content of key national-level policy documents are summarized in Table 2.

### 3.1. Policy Landscape of Rare Disease Protection in China

#### 3.1.1. Constraints of the Basic Medical Insurance (BMI) System

To alleviate the financial burden on patients with rare diseases, over 120 orphan drugs have been included in the NRDL (2024 Edition), covering more than 60 rare conditions, based on diseases listed in the First and Second Lists of Rare Diseases [20,28,32]. A detailed list of included drugs is shown in Appendix A. However, from a payment structure perspective, the current BMI system still falls short in providing adequate coverage for treatments of rare diseases.

**Figure 2:**
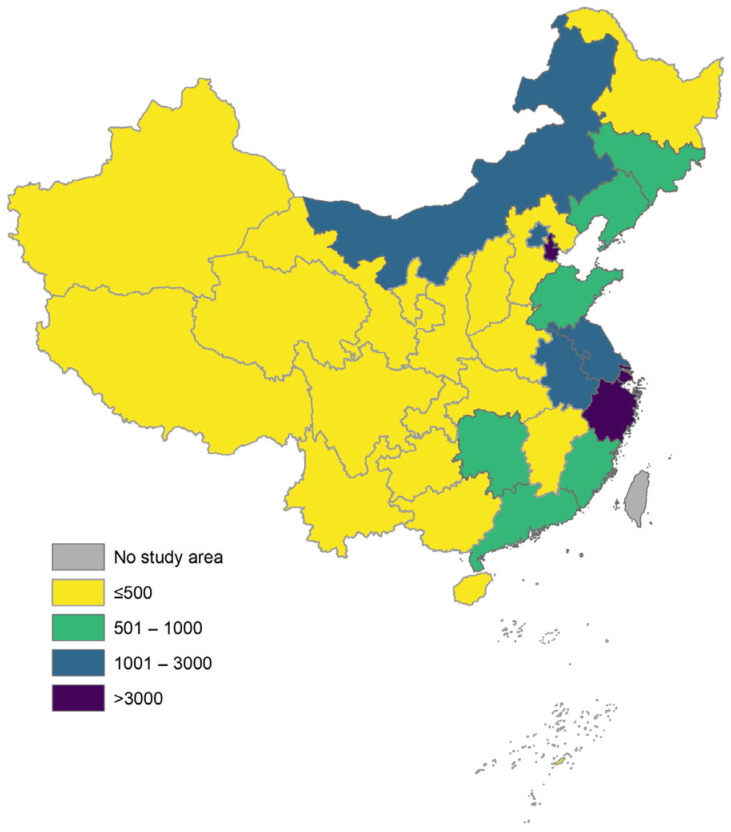
Distribution of annual reimbursement caps for outpatient services under urban and rural resident basic medical insurance (URRBMI) in 31 provincial-level administrative centers. Note: The reimbursement caps shown in the map are represented in CNY.

**Figure 3:**
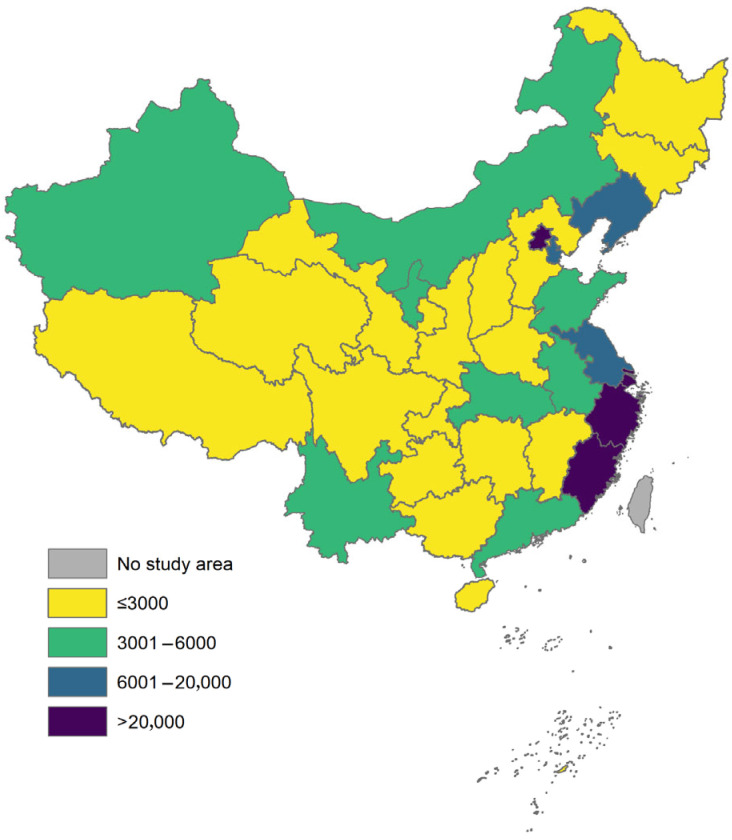
Distribution of annual reimbursement caps for outpatient services under urban employee basic medical insurance (UEBMI) in 31 provincial-level administrative centers. Note: The reimbursement caps shown in the map are represented in CNY.

On the one hand, reimbursement for rare diseases under the healthcare system is generally divided into outpatient and inpatient categories; yet, most rare disease treatments are conducted in outpatient settings. However, the reimbursement limits for outpatient care across various regions are typically low, making it challenging to cover the long-term, ongoing treatment needs of patients with rare diseases. According to our analysis of outpatient reimbursement policies in 31 provincial-level cities (see Figure 2 and Figure 3), the reimbursement cap under the urban and rural resident basic medical insurance (URRBMI) is below RMB 500 in most regions, which is insufficient to cover the ongoing medication expenses required for treatments of rare diseases. Although the urban employee basic medical insurance (UEBMI) generally sets higher caps, most cities still set the limits below RMB 3000, which remains inadequate to cover the annual treatment costs of orphan drugs, often amounting to tens or even hundreds of thousands of yuan. Furthermore, many patients with rare diseases are children or non-working individuals who typically fall under the URRBMI scheme, which generally offers lower reimbursement rates and limited coverage. Combined with strict reimbursement caps, this results in high out-of-pocket expenses for patients.

Existing studies based on drug simulation calculations for rare disease medications (such as recombinant human coagulation factor VIIa for hemophilia and eculizumab for myasthenia gravis) indicate that, on average, out-of-pocket expenses for outpatient patients exceed 90% in 31 provincial-level administrative centers [33,34]. This further suggests that, although healthcare insurance covers some treatment costs, the reimbursement limits for outpatient visits remain insufficient to alleviate the substantial financial burden on patients with rare diseases. As a result, patients face substantial economic pressure, leading to a system structure where high medical costs are shifted to families.

On the other hand, China’s current healthcare system operates under a “centralized policymaking and decentralized implementation” model, where the central government sets guiding principles and reimbursement catalogs. In contrast, local healthcare authorities adopt policies based on local funding capacity and administrative feasibility [35]. Although this governance model allows for flexibility, in the context of rare diseases characterized by diagnostic complexity, specialized treatment pathways, high drug prices, and small patient populations, effective implementation depends heavily on local administrative capacity. In 2021, the NHSA and the MOF jointly issued the Opinions on Implementing a Standardized Healthcare Benefits Catalog, encouraging local governments to include certain rare diseases under outpatient special disease or chronic disease management schemes [23]. Nevertheless, due to regional disparities in fiscal capacity, the inclusion of diseases, reimbursement scope, and benefit levels varies significantly, leading to substantial geographic inequities in access to care for patients with rare diseases [36]. Even when rare diseases are formally included in local outpatient special disease or chronic disease schemes, effective reimbursement rates are often limited by fiscal constraints, thereby failing to provide adequate financial protection.

According to an existing study on the inclusion of rare diseases in the outpatient chronic and special disease lists of 300 prefecture-level cities across China, the data show that only 24 rare diseases are covered by local medical insurance systems, with most cities covering fewer than four rare diseases [37]. Taking hemophilia as an example, the average annual treatment cost for this condition is RMB 188,100. Nationwide, 233 cities have included hemophilia in their urban outpatient chronic disease and special disease coverage; however, 87.12% of these cities’ medical insurance plans have actual reimbursement rates below 50%. This further indicates that, even when rare diseases are included under local outpatient special disease or chronic disease management schemes, the reimbursement levels often remain insufficient to meet actual treatment needs.

From an institutional perspective, China’s BMI is designed to provide “broad coverage and basic protection” for its 1.4 billion population. Given the limited financial pool, the system faces a critical trade-off between allocating resources to high-cost rare disease patients and ensuring coverage for more prevalent, lower-cost conditions. With increasing numbers of new, high-priced drugs entering the market, it becomes unsustainable for the BMI fund to fully cover such treatments without compromising overall system equity. Therefore, high-cost therapies—including orphan drugs—are often excluded from the NRDL.

Viewed through the lens of risk management theory, the current system tends to concentrate “risk retention” rather than distributing it across a broader social safety net. High-cost rare disease populations are excluded mainly from effective risk-sharing mechanisms, resulting in the individualization of what are, in essence, collective health risks. The resulting pattern—frequent catastrophic health expenditures—places enormous strain not only on households but also on the capacity of the insurance fund to fulfill its redistributive and risk-pooling functions. This configuration reveals a more profound structural vulnerability in the financial design of China’s rare disease insurance framework, particularly in its capacity to protect the high-risk group.

#### 3.1.2. Limited Effectiveness of Supplementary Security Mechanisms

Currently, key policy documents issued by the State Council—such as the Opinions on Deepening the Reform of the Medical Security System and Opinions on Improving the Medical Insurance and Assistance System for Catastrophic Diseases issued by the General Office of the State Council—emphasize the need for a multi-tiered medical security system for rare diseases [22,24]. These policies advocate that supplementary protection mechanisms, including commercial insurance, government-backed inclusive health insurance, and social philanthropy, should play a role in “risk succession” and serve as a “buffer of flexibility” when the BMI system reaches its functional limits. However, the current institutional structure of China’s supplementary security system has not effectively absorbed the high medical costs associated with rare diseases, resulting in a systemic discontinuity, or “gap in institutional relay.”

Although commercial health insurance schemes (including semi-public policy-backed plans such as Huiminbao) have been actively exploring pathways for coverage of rare diseases, their operating logic is fundamentally misaligned with the characteristics of these diseases. These plans are highly susceptible to adverse selection, exhibiting strong demand from individuals with pre-existing conditions and weak enrollment incentives among healthy populations [38,39,40]. This imbalance seriously undermines the actuarial sustainability of commercial insurers.

While commercial health insurance plans often claim to cover both reimbursable and non-reimbursable items under the NRDL, including high-cost medications, many still exclude orphan drugs or genetically inherited conditions from their benefit packages [41]. In cases where pre-existing conditions are accepted, insurers often impose restrictive measures, such as higher deductibles or lower reimbursement rates, to mitigate financial risk. For instance, the “Puhui Health Insurance” in Beijing explicitly excludes “genetic diseases, congenital malformations, and chromosomal abnormalities” in its terms, providing no coverage for individuals diagnosed with these conditions prior to enrollment. Similarly, “Huhui Insurance” in Shanghai sets differentiated reimbursement rates for specialty drugs: non-pre-existing condition groups can receive a 70% reimbursement, while those with pre-existing conditions are reimbursed only 30%. These examples illustrate how commercial insurers, through exclusion clauses and differentiated reimbursement structures, may avoid risks, thereby significantly weakening the actual level of protection for patients with rare diseases and curtailing the potential for commercial insurance to serve as a supplementary option within the rare disease coverage framework. According to the 2022 China Insurance Industry Social Responsibility Report issued by the Insurance Association of China [42], the total commercial health insurance payout in 2022 was RMB 360 billion, accounting for only 5% of national health expenditure, 7% of direct medical spending, and 14.7% of the BMI fund expenditure.

Social assistance programs administered by civil affairs departments are primarily targeted at socioeconomically disadvantaged populations. Due to the earmarked nature of these funds, such programs are not equipped to meet the needs of high-cost patient populations, including those with rare diseases. Although rare disease-focused philanthropic organizations demonstrate a strong commitment to providing support, their limited fundraising capacity constrains their ability to deliver substantial and sustained financial assistance.

In addition to nationally oriented mechanisms, several provincial governments have sought to establish dedicated rare disease funds as supplementary measures to address the high costs associated with rare disease treatments. For instance, provinces such as Zhejiang and Jiangsu have allocated a fixed annual amount (e.g., 2 CNY per capita) from their critical illness insurance pools to subsidize high-cost orphan drugs that are not included in the NRDL [35]. However, these financing models remain embedded within the existing BMI pooling framework and rely primarily on intra-pool fund reallocation rather than the creation of independent financing channels. As a result, they continue to face the structural limitations of the BMI system in terms of revenue generation and benefit distribution, thereby limiting their capacity to effectively close coverage gaps for rare diseases.

Taken together, the landscape of rare disease coverage in China remains highly dependent on the BMI system, lacking diversified financing channels and flexible benefit structures. This results in high levels of retained risk, increasing out-of-pocket burden, and a rigid institutional boundary. From the perspective of risk management theory, establishing a multi-tiered, multi-payer collaborative risk-sharing system is crucial for effectively mitigating the systemic health risks faced by populations with rare diseases.

### 3.2. Misalignment Between Coverage Timing and Disease Characteristics

Genetic mutations predominantly cause rare diseases. They encompass a broad spectrum of conditions, are characterized by complex clinical pathways, and often manifest early in life with a high rate of disability [43]. According to a nationwide survey conducted by the Chinese Organization for Rare Disorders (CORD) involving 20,804 patients, 42% of respondents had experienced misdiagnosis, and 15.5% waited between one and 5 years before receiving an accurate diagnosis. On average, the time to diagnosis was 4.26 years, with some cases taking as long as 44 years [44] (pp. 16, 17, 20). Given the diagnostic delays and the high cost of treatment, limiting health coverage to post-diagnosis interventions often results in missed opportunities for early treatment. Many children only receive interventions when their disease has progressed to a moderate or severe stage, which compromises clinical outcomes and significantly reduces the cost-effectiveness of both public insurance and fiscal subsidies.

Current national policies have limited coverage of early services such as prenatal genetic screening, genetic testing, and counseling. Consistent with this, existing literature suggests that China’s rare disease reimbursement framework tends to focus on expenses incurred after diagnosis, especially pharmaceutical costs [33,45,46]. This means that the current insurance mechanism often uses “diagnosis” as the entry point for reimbursement eligibility, neglecting preventive measures in the prenatal and early life stages.

From the perspective of risk management theory [16], this institutional design reveals a critical absence of risk identification mechanisms and a weakened capacity for risk avoidance. In addition, the health capital model highlights the inefficiency of this structure, as it fails to support early-life interventions, thereby hindering the optimal accumulation of health capital and reducing the marginal return on healthcare investments. For high-cost treatments, the lack of a stable reimbursement mechanism increases the risk of treatment discontinuation, which may lead to disease recurrence and, in turn, escalate public healthcare expenditures. As a result, this structural mismatch between the timing of financial protection and the natural course of disease progression not only compromises patient outcomes but also undermines the efficiency and sustainability of the broader healthcare system.

These design limitations—spanning payment structures, inclusion criteria, intervention timing, and governance architecture—have collectively led to a persistent imbalance between high risk and insufficient response capacity. From a theoretical standpoint, such deficiencies reveal breakdowns at every stage of the risk management chain: risk identification is delayed, risk transfer is weak, and risk control remains underdeveloped. Furthermore, the absence of health capital investment logic exacerbates these failures, as early-stage health interventions—those with the most excellent marginal returns—are underutilized. Addressing these institutional mismatches is essential for shifting from reactive, post-diagnosis compensation toward anticipatory, front-loaded interventions and building a more coherent and responsive rare disease security system.

## 4. Discussion

### 4.1. International Models and Localization Challenges

International practice has shown a growing trend toward layered insurance frameworks, which combine basic universal coverage with supplementary schemes targeted at specific high-risk groups. This structure enables stratified and multi-actor participation, facilitating the institutional transfer and systematic sharing of health risks related to rare diseases.

Germany, under its unified social health insurance system, imposes an annual cap on individual out-of-pocket medical expenses, limiting them to no more than 2% of a household’s yearly income, even for patients with rare diseases [47]. This universal mandatory coverage, combined with a clearly defined financial protection threshold, mitigates the impact of income disparities on treatment access and significantly enhances equity in rare disease protection. Turkey adopts the “rule of rescue,” prioritizing the preservation of life and the principle of health equity regardless of treatment cost. This ensures orphan drug accessibility and horizontal equity for all eligible rare disease patients [48]. The United States relies on a mature commercial health insurance market, supplemented by federal programs such as Medicaid and the incentives provided under the Orphan Drug Act, which prohibits commercial insurers from denying coverage to rare disease patients. With only a modest premium increase, this framework achieves near-universal coverage (about 99% of patients), combining market depth, legal guarantees, and tailored benefits to ensure both fairness and sustainability [14,15]. The United Kingdom funds its National Health Service (NHS) through general taxation and, in 2011, established the Cancer Drugs Fund (CDF) to finance anti-cancer medicines not yet evaluated or approved by the National Institute for Health and Care Excellence (NICE) [49]. Directly funded and administered by the central government, the CDF ensures nationwide uniformity in standards and resource allocation. Belgium operates a “Special Solidarity Fund” to provide last-resort coverage for an extremely small number of ultra-high-cost cases, supported by rigorous case review and strict fiscal caps to target resources precisely while preserving system sustainability [10]. Australia operates the Life Saving Drugs Program (LSDP) to provide targeted support for high-cost rare disease drugs not included in the Pharmaceutical Benefits Scheme (PBS) [11]. Fully financed from the federal budget and centrally approved, the LSDP ensures that even very small patient populations can access life-saving treatments, while preventing disparities in coverage between states and territories. Japan enacted the Act on Medical Care for Patients with Intractable Diseases in 2014, which explicitly includes certain rare diseases, thereby establishing the legal framework for rare disease prevention, designated conditions, and a dedicated health insurance mechanism. Since 2015, the centrally managed Medical Care Program for Specific Diseases has been financed primarily through consumption tax revenue, offering up to 80% reimbursement for covered treatments. Currently encompassing 333 diseases and benefiting approximately 940,000 patients [12], the program alleviates household financial burdens and equalizes coverage levels across regions through centralized fiscal redistribution. Taiwan (China) promulgated the Rare Disease Prevention and Orphan Drug Act in 2000, with Article 33 authorizing the central government to allocate budgetary resources to cover rare disease treatments excluded from the National Health Insurance scheme. To ensure stable funding and long-term sustainability, Taiwan earmarks part of the tobacco health welfare surcharge, levied under the Tobacco Hazards Prevention Act, to the Rare Disease Prevention and Control Fund [13], thereby bridging coverage gaps in basic health insurance and creating a permanent, earmarked mechanism within the public finance framework. To systematically compare the institutional logic behind rare disease coverage across countries, Table 3 organizes key features of each model, focusing on their basic coverage structure, supplementary mechanisms, and financing channels.

However, these models are embedded in distinct socio-economic and governance contexts, which pose substantial barriers to direct transplantation into China.

First, given China’s large population base and uneven fiscal capacity across regions, the current BMI system—designed primarily to “ensure basic needs”—lacks the institutional orientation and resource capacity to support the comprehensive, high-coverage, and government-led welfare models seen in countries such as Germany and Turkey.

Second, the U.S. model relies on a well-developed commercial insurance market, a robust credit infrastructure, and sophisticated risk management, with public programs such as Medicaid serving as complements. In contrast, China’s commercial insurance penetration remains low, and its mechanisms for actuarial risk assessment and long-term claims management are still underdeveloped.

Third, models such as the UK’s CDF, Australia’s LSDP, Japan’s Medical Care Program, and Taiwan’s Rare Disease Prevention Fund rely on concentrated fiscal authority and statutory earmarking—features difficult to replicate in China’s “central guidance–local implementation” structure, where no le-gal category for a national rare disease fund exists and no permanent, earmarked central budget is in place. Belgium’s case-by-case Special Solidarity Fund, while effective in a small-population setting, would be operationally unfeasible in China, where even ultra-rare diseases involve large absolute case numbers.

Against this backdrop, rather than overstretching existing BMI systems, a more institutionally endogenous and risk-sensitive approach would be to establish a disease-specific insurance scheme targeting high-cost, high-risk rare diseases. Such a policy-guided mechanism could promote voluntary enrollment, enable targeted subsidies, and create a relatively independent, transparent, and financially sustainable subsystem for rare disease protection, while gradually building toward a more nationally coordinated framework.

### 4.2. Institutional Insights from Risk Management and Health Capital Theory

According to risk management theory [16], institutional design is expected to emphasize preemptive interventions before risks materialize into actual losses. Two core mechanisms—risk avoidance and loss control—form the foundation of this approach.

In the context of rare diseases—approximately 80% of which are hereditary—preconception carrier screening, prenatal genetic testing, and genetic counseling serve as practical instruments of risk avoidance, especially in genetically inherited conditions. These interventions enable the early identification of high-risk pregnancies and provide informed decision-making support, thereby reducing the likelihood of births with severe congenital conditions. This not only improves population-level health outcomes but also alleviates long-term financial burdens on public insurance and welfare systems.

When risks cannot be entirely avoided—for example, if cases remain undetected or families choose to proceed with pregnancy—loss control becomes essential. Prompt diagnosis and timely therapeutic intervention, while not eliminating risk, can reduce disease severity, delay clinical deterioration, and improve long-term outcomes, thereby enhancing both patient well-being and health system sustainability. This is especially true for early-life interventions, which address conditions promptly and maximize the potential for long-term benefits.

Ethically, children as a vulnerable population have their rights to life and health accorded high moral priority in public policy. Research shows broad-based public support for using fiscal resources to support treatments for rare diseases in children, with earlier interventions garnering even greater policy endorsement [12,50].

Therefore, initiating protection during the earliest stages of life reflects the core logic of the two theoretical approaches, which emphasize preemptive action and early investment. When coupled with ethical and policy consensus regarding child health rights, this rationale provides a strong foundation for developing anticipatory, early-stage rare disease protection mechanisms.

### 4.3. Policy Innovation: The Dedicated Insurance Scheme for Rare Diseases (DISRD)

The theoretical analysis emphasizes the importance of shifting from passive compensation to proactive risk management, particularly through early intervention and life-course coverage. However, China’s current healthcare insurance system still faces fundamental challenges in its ability to provide comprehensive, proactive coverage that addresses risks before they fully materialize. The gap between theoretical demands and institutional capabilities highlights the urgent need for innovative policy solutions to address the unique requirements of rare disease coverage.

Evidence from multiple stakeholders further substantiates this necessity. At the national level, interviews with patient organizations indicate that 82.1% of respondents recommend establishing a dedicated rare disease insurance scheme to cover expenses not reimbursed by basic medical insurance [51]. Surveys of physicians and interviews with experts likewise identify such dedicated insurance as the most promising approach to addressing the high costs of orphan drugs, while stressing the critical importance of early screening and diagnosis for improving treatment outcomes [52]. At the local government level, regions such as Gansu, Beijing, Foshan, and Qingdao, among others, have explored diversified rare disease protection arrangements to subsidize high-cost treatments not included in the national reimbursement list, reflecting a proactive fiscal and policy commitment to rare disease protection [35,41]. This dual foundation of theoretical and practical evidence not only underscores the necessity of an innovative institutional design but also provides a robust empirical and conceptual basis for the proposed Dedicated Insurance Scheme for Rare Diseases (DISRD).

Building on this foundation, we propose DISRD as a forward-looking institutional design that integrates risk avoidance, loss control, and sustained protection through staged coverage. Anchored in the theoretical framework, this section systematically outlines the six core operational elements of DISRD: (1) financing mechanisms, (2) enrollment and risk identification, (3) benefits design and claims processes, (4) renewal and adverse selection controls, (5) disease and drug selection, and (6) implementation considerations and pilot pathway. These elements collectively embody the principles of equity, fiscal sustainability, and practical feasibility.

**Figure 4:**
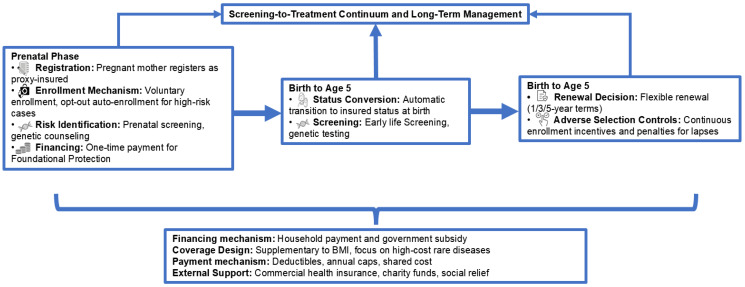
The lifecycle flowchart of the DISRD system: from screening to treatment continuum and long-term management.

To further clarify how these components work throughout an individual’s lifecycle, we have included a flowchart that visually summarizes the system from prenatal screening to long-term management. As shown in Figure 4, this illustration provides a clear overview of the system’s key stages and their interconnections, simplifying the complex process and enhancing understanding of the dynamic interactions between different operational elements.

#### 4.3.1. Financing Mechanism

This system design fully considers the hierarchical responsibility structure of “central guidance, local coordination” in the operation of China’s healthcare financing system, aiming to achieve an effective balance between institutional embedding and fiscal adaptability. Operationally, the scheme would be hosted within the administrative and information infrastructure of the existing basic medical insurance system, while maintaining a financially independent special account with separate management and accounting. This arrangement ensures clear segregation of sources. Existing literature has also pointed out that [35,37,53], to cover high-cost rare disease medications not currently included in the BMI, it is necessary to establish a separate funding pool outside the healthcare insurance system, providing a differentiated institutional protection pathway.

The financing mechanism of the DISRD system adopts a dual-track structure, referring to a two-part funding model that combines household contributions with local government matching subsidies, aiming to ensure both the broad accessibility of the system and its fiscal sustainability. The household contribution component is based on principles of voluntariness, low entry thresholds, and non-mandatory participation, aiming to enhance social acceptance and increase enrollment coverage. To further promote equity and progressive redistribution, a tiered subsidy mechanism is proposed. Drawing on Taiwan’s Rare Disease Prevention and Treatment and Drug Act [13], which provides full subsidies to low-income populations, and incorporating the concept of ‘tiered subsidy’ from China’s urban and rural residents’ healthcare insurance system [54,55], the proposal suggests offering full or partial premium subsidies to vulnerable groups, such as those identified by the civil affairs system as being in poverty or requiring special care. This approach ensures that individuals are not excluded from the system due to financial hardship.

The government subsidy component is proposed to follow the well-established model in the urban and rural residents’ healthcare insurance system [54], where the central government sets a baseline and local governments have the flexibility to increase subsidies. Specifically, the central government will establish a unified financial subsidy standard for the entire country and implement a tiered subsidy policy based on regional fiscal capacities. West and central regions will receive higher levels of support, while the more economically developed eastern regions will bear a greater share of the financial responsibility. Local governments will have the flexibility to increase the central subsidy according to their actual circumstances, using resources from local public budgets, public health special funds, or other financial channels, thereby strengthening local healthcare capacity. For regions with weaker fiscal capacities, the central government may provide targeted support through equalization transfer payments to help balance the level of protection across the country. Regarding the central government’s subsidy funding arrangements, in the early stages of the system, priority can be given to utilizing existing financial channels, such as public health special funds, general public budget surpluses, and welfare lottery funds. These funds have already been widely applied in existing significant disease relief and rare disease assistance policies, making them practically feasible [41,56].

From an international perspective, Japan’s “Medical Care Program for Specific Diseases” [12,57], which is centrally managed and funded through a unified consumption tax, achieves shared risk-bearing and fund allocation nationwide, effectively improving the system’s financial predictability and regional equity. This experience provides valuable insights for enhancing the fiscal stability and system resilience of the DISRD in the future.

However, directly transplanting such a centralized funding model to China is currently infeasible due to institutional and operational constraints. On the one hand, China’s existing health insurance financing framework emphasizes “central guidance with local pooling,” with pronounced disparities in local fiscal capacity; on the other hand, the enactment of unified legislation and the establishment of dedicated tax instruments remain subject to high institutional thresholds. Consequently, the immediate adoption of a national-level pooling system is not yet feasible.

In view of these considerations, DISRD should take the “local coordination, central guidance” framework as a pragmatic starting point in the initial stage. As the institutional framework matures, the establishment of a cross-regional mutual assistance mechanism can serve as a transitional arrangement, with the ultimate goal of achieving a higher level of fiscal integration across the country. As the system matures and the “Healthy China 2030” blueprint’s directive to incorporate unhealthy product tax revenues into public health spending is gradually implemented, some earmarked revenues (such as tobacco and sugary beverage taxes) could be incorporated into central subsidies [58]. This would provide a more resilient and sustainable funding base for the program. This gradual approach would not only maintain regional adaptability and operational flexibility but also enhance cross-regional risk sharing and budget stability

#### 4.3.2. Enrollment and Risk Identification

To implement the concept of proactive risk control in rare disease protection, this study proposes the establishment of a “Rare Disease Specialized Insurance” system, starting from the pregnancy stage, and outlines an operationally feasible framework. Unlike the “Huiminbao”, a government-guided commercial insurance product typically offered by private insurers, the rare disease specialized insurance plan proposed in this study is not profit-driven. Instead, it is designed as a targeted public policy tool that provides specific fiscal support, clear eligibility criteria, and long-term planning objectives tailored to the unique burden of rare diseases.

In terms of enrollment pathways, the system introduces a “pregnancy pre-coverage mechanism,” shifting the coverage responsibility to the prenatal stage, thereby enabling early identification of risks and intervention. The National Health Commission’s Birth Defect Prevention and Control Capacity Building Plan (2023–2027) [59] has already explicitly incorporated the three-tiered congenital disability prevention system, encompassing pre-conception, prenatal, and neonatal stages, as part of the national maternal and child health policy. It emphasizes the need to continuously improve the service chain for neonatal disease screening, diagnosis, and treatment, and to promote early screening, diagnosis, and treatment. This initiative confirms the feasibility of incorporating screening and early intervention into the design of the rare disease protection system, and provides a direct policy foundation for shifting interventions upstream.

Leveraging the existing maternal and child health information platform, a traceable enrollment mechanism will be established, with grassroots maternal and child healthcare institutions providing early enrollment notifications and policy guidance during the early stages of pregnancy. Pregnant women, as “potential insured representatives,” will complete registration and payment. After the birth of the infant, the coverage will automatically transition to the formal insured status, ensuring seamless coverage. This model encompasses the entire process, from prenatal screening and birth diagnosis to early treatment and long-term management, forming an integrated lifecycle protection system of “screening—diagnosis—treatment—management—coverage.”

To enhance the coverage of high-risk groups under the voluntary enrollment principle, this study proposes an “opt-out” mechanism for individuals identified as high-risk through medical screening (such as those with abnormal prenatal screening results or neonatal metabolic disorders). Specifically, these individuals will be automatically included in the protection system unless their guardians explicitly opt out, in accordance with both legal and ethical standards. Following automatic enrollment, high-risk individuals will be able to directly access the aforementioned tiered subsidy mechanism (as detailed in Section 4.3.1). For example, high-risk infants identified as low-income or in special difficulty by the civil affairs system will benefit from subsidy policies designed for low-income groups, receiving full or partial financial support from the government, thus ensuring the system’s targeted assistance and progressive redistribution objectives.

Through the integration of this “risk identification—automatic enrollment—classified funding” pathway, the medical screening and healthcare insurance registration systems can be effectively connected, promoting the establishment of an efficient execution logic across the entire process of identification, enrollment, and funding. This approach respects family autonomy while significantly reducing the risk of coverage gaps, thereby enhancing the fairness and inclusiveness of the system.

#### 4.3.3. Benefit Design and Claims Process

In the benefit design, DISRD emphasizes complementing the functions and responsibilities of basic medical insurance by implementing a “differentiated compensation” strategy. For rare disease drugs already included in the insurance catalog, the specialized insurance will cover the remaining costs after basic medical insurance reimbursement, thereby avoiding redundant coverage. For high-value, specialized drugs that have not yet been included in the insurance catalog but are selected through an evaluation system, reimbursement rates and annual caps will be determined based on the fund’s capacity to manage payment risks and ensure equity and accessibility.

In terms of payment mechanisms, studies have highlighted the significant role of incorporating reasonable risk retention mechanisms into insurance design to control healthcare costs and promote the rational use of medical services [60,61]. Accordingly, this study recommends introducing risk retention mechanisms by setting deductibles, co-payment ratios, and caps on screening costs. These measures would encourage insured families to bear a reasonable share of the financial responsibility, enhance their health management awareness, and prevent system misuse and waste of resources. Furthermore, considering that some high-value drugs may lack sufficient evidence in the initial market launch, we recommend implementing a dynamic adjustment mechanism linked to real-world data [62,63]. This will encourage pharmaceutical companies to participate in patient tracking studies, share efficacy data, and use this as a basis for adjusting payment standards and reimbursement strategies. As the system matures, pay-for-performance mechanisms could be further explored to improve fund operation efficiency, enhance corporate participation, and strengthen collaboration.

To improve the rare disease risk protection system, DISRD should also proactively collaborate with civil affairs, medical aid, and charity foundations as social resources. Particularly for high-cost treatments for rare diseases that are not fully covered by basic medical insurance and specialized insurance, “filling-in support” should be provided, ensuring full coverage from institutional protection to public assistance, thereby enhancing system resilience and social equity.

In terms of the claims process, the DISRD needs to establish a standardized and traceable claims handling mechanism that ensures seamless integration with the existing healthcare insurance system. Drawing on the experience of Australia’s PBS-LSDP linkage mechanism, the DISRD can adopt a “primary healthcare insurance platform with modular expansion” architecture to embed into the existing information system. This would unify data flow, reimbursement flow, and review processes, thereby avoiding redundant claims, fragmented information, and administrative inefficiencies, ultimately reducing management costs.

#### 4.3.4. Renewal and Adverse Selection Control

Regarding the payment mechanism, it is recommended to implement a hybrid model consisting of an initial lump-sum payment followed by periodic renewal payments (e.g., annual, three-year, or five-year cycles) to achieve a dynamic balance between early fund accumulation and later risk distribution. The initial payment phase spans the period from pregnancy to age 5, a critical window during which rare diseases exhibit a high incidence of both morbidity and mortality. According to Orphanet data, approximately 30% of children with rare diseases die before the age of 5, making it a crucial phase for screening, diagnosis, and early intervention. This stage can be defined as the “basic coverage period”, where the lump-sum payment ensures coverage of essential medical services and leaves reserves to offset future claims, thereby enhancing the system’s early operational stability.

After age 5, the system enters the “renewal choice period”, where families can decide whether to renew coverage based on their risk and coverage needs. However, considering the high treatment costs, uncertainty, and recurrence risks associated with rare diseases, adverse selection may occur during the renewal process. Healthier families may opt out of the insurance, while higher-risk families may continue, thus increasing the fund’s claim pressure and compromising the system’s sustainability. To mitigate this, we propose drawing on the current BMI model to introduce mechanisms that encourage continuous enrollment and impose penalties for interruptions in coverage [55]. This would reduce the likelihood of adverse selection. Specifically, the system could implement incentives for continuous enrollment, such as increasing reimbursement rates or reducing renewal premiums, to encourage families to maintain uninterrupted coverage. At the same time, penalty mechanisms for coverage interruptions, such as waiting periods or additional payments upon resumption of coverage, would help address adverse selection and ensure the long-term sustainability of the system.

Furthermore, various payment cycle options can be provided during the renewal phase, such as annual payments, three-year payment plans, or five-year payment plans, to accommodate different family preferences. This approach would enhance the fund’s stability and increase renewal rates. Empirical studies have shown that policy flexibility in renewal conditions and cycle choices is significantly positively correlated with the willingness of enrollees to renew. When participants can adjust renewal periods according to their needs, the likelihood of continued enrollment increases significantly [64,65,66]. This provides empirical support for introducing multi-cycle renewal mechanisms in DISRD, highlighting its practical feasibility and policy value.

#### 4.3.5. Disease and Drug Selection

##### Disease Selection

Currently, China lacks an official epidemiological definition of rare diseases [56]. Instead, the regulatory framework defines the scope of rare disease management by publishing official lists of diseases [6]. In the initial phase of the DISRD, the coverage scope may be defined based on “The First Catalog of Rare Diseases (2018)” and “The Second Catalog of Rare Diseases (2023),” jointly issued by the health authorities, which collectively include 207 conditions [20,28].

Within this defined scope, further prioritization of diseases for coverage can be conducted through a multidimensional evaluation process. Criteria such as disease severity, economic burden, availability of effective treatment, and estimated patient population should be considered. A scoring and expert consensus approach may be applied to prioritize diseases [67]. Additionally, a dynamic adjustment mechanism involving health authorities, medical insurance agencies, clinical experts, and patient organizations should be established to ensure periodic and evidence-based updates to the list. This would enhance the adaptability and foresight of the insurance policy.

##### Drug Selection

There is no standardized definition for drugs for rare diseases in China [41], and the term does not fully align with the internationally recognized concept of “orphan drugs.” Rare disease drugs in China can be broadly categorized into two types: (1) “general-purpose drugs,” which are used to treat a range of conditions including rare diseases—for example, glucocorticoids for 21-hydroxylase deficiency—most of which have already been included in the NRDL or made accessible through price negotiations and centralized procurement; and (2) “specialized drugs,” which are indicated exclusively for specific rare diseases and are typically high-cost. Due to the substantial price gap and lack of consensus between pharmaceutical companies and the NHSA during pricing negotiations, these drugs often remain uncovered, resulting in significant financial burdens for patients. Examples include eculizumab for atypical hemolytic uremic syndrome, paroxysmal nocturnal hemoglobinuria, and satralizumab for neuromyelitis optica spectrum disorder. These disparities in drug classification and pricing structure have created a notable coverage gap within the existing reimbursement system for high-cost, specialized, and rare disease drugs. Against this backdrop, the DISRD must account for drug typologies and the realities of the current payment system to develop a differentiated coverage mechanism that promotes efficient resource allocation and seamless policy integration.

Given the insurance fund’s limited financial capacity, providing full reimbursement for all high-value, rare disease drugs is not feasible in the short term. Therefore, establishing a transparent, evidence-based, and dynamically adjustable drug selection mechanism is crucial for sustainable resource allocation and risk management. To this end, developing a multidimensional drug value assessment framework grounded in the principles of evidence-based medicine and health economics is recommended, thereby enhancing the scientific rigor, systematic structure, and policy relevance of the selection process.

Specifically, drug evaluation should focus on the following six core dimensions [68,69,70,71,72]:

1. Disease Burden: Systematically assess the disease’s impact on patients’ quality of life, labor capacity, and the consumption of social care resources to identify the overall burden it imposes.
2. Clinical Effectiveness: Evaluate the drug’s therapeutic advantages within the existing treatment landscape, its irreplaceability, and its capacity to address previously unmet medical needs [73].
3. Quality of Evidence: Examine the robustness of clinical trial data, real-world evidence, expert consensus, and clinical practice guidelines to ensure that selection decisions are based on high-quality evidence.
4. Economic Consequences: Conduct a comprehensive analysis of the drug’s direct costs, associated healthcare expenditures (e.g., hospitalization, diagnostic testing), and non-medical costs (e.g., productivity loss, caregiving expenses), as well as their impact on both insurance funds and overall societal burden.
5. Innovative Value: Considering the novelty of the drug mechanism, its ability to fill clinical gaps, and designation as a “breakthrough therapy,” with corresponding weighted scoring [74].
6. Accessibility and Added Value: Examine factors such as whether the drug has been approved for marketing in China, the availability of clinical trials involving Chinese populations, and whether global launch timelines are aligned (with a time difference not exceeding 6 months).

Given that drug value assessment necessitates the comprehensive consideration of clinical, economic, innovation-related, and other pertinent factors—and recognizing that different stakeholders may hold divergent value orientations—it is crucial to establish selection criteria that balance multiple standards and incorporate diverse perspectives. Accordingly, this study recommends the application of Multi-Criteria Decision Analysis (MCDA). This methodology has been increasingly employed in orphan drug evaluations [71,75,76] to support scientific decision-making across multiple, sometimes conflicting, dimensions.

#### 4.3.6. Implementation Considerations and Pilot Pathway

In the early stage of implementation in China, the DISRD may face limited policy support at the subnational level, as some local governments could be cautious about adopting a new scheme perceived as fiscally burdensome or administratively complex. From a legal perspective, the current statutory framework, including the Social Insurance Law, has yet to establish a dedicated legal category or a stable and sustainable funding source for a national rare-disease benefit (e.g., Taiwan’s use of a tobacco surcharge), which constrains the legitimacy and feasibility of earmarked fiscal arrangements. Administratively, the implementation process may be hindered by the lack of experience among local insurance agencies in rare disease identification, claims review, and payment management, as well as by limited interdepartmental coordination and weak interoperability across health, insurance, and civil affairs information systems—factors that may undermine efficiency and equity in the initial phase.

Given these multidimensional challenges, a controlled and incremental pilot pathway would not only help mitigate the political and fiscal risks of policy innovation but also allow a limited-scale assessment of fiscal sustainability and administrative capacity, thereby laying the institutional and technical foundation for nationwide rollout.

It is recommended to conduct zonal pilots with progressive expansion: select one province each from the eastern, central, and western regions to conduct provincial-level pooling pilots, thus covering diverse economic, institutional, and administrative contexts to enhance representativeness and robustness. The pilot period is recommended to be 5 years, with funding drawn from the existing fiscal channels described in Section 4.3.1, earmarked for exclusive use and allowing retention of surpluses for subsequent years. A fiscal risk early-warning mechanism should be introduced to test the viability of existing funding arrangements under different economic conditions, while on the administrative and technical side, modular interoperability between health, medical insurance, and civil affairs information systems should be promoted alongside capacity-building for implementing agencies. During the pilot, an annual monitoring and evaluation mechanism should be established, focusing on core indicators such as fund balance stability (e.g., solvency ratio), enrollment coverage, service accessibility, and the reduction in patients’ out-of-pocket spending. Evaluation results should trigger adaptive adjustments, including dynamic revision of disease and drug lists, subsidy levels, and, where necessary, financing structures and process redesign. Once the pilot meets predetermined performance thresholds, expansion should follow a sequence of “priority to regions with similar conditions, then gradual inclusion of regions with greater differences,” and—upon achieving data interoperability and standard harmonization—advance toward higher-level fiscal integration and cross-regional risk pooling.

### 4.4. Multi-Tiered Protection Framework for Diagnosed Patients

For individuals already diagnosed with rare diseases, establishing a comprehensive, clearly defined, and structurally sound multi-tiered protection system is essential to address the high-frequency and high-cost medical risks they face. From a risk management perspective [16], responses to realized risk events should not rely on a single institutional mechanism. Instead, a multi-layered and coordinated risk dispersion framework is required to enhance the overall system’s flexibility and long-term sustainability.

In terms of institutional design, the government should serve as the central actor in system architecture and resource allocation [22,58,77]. It must promote collaborative governance among public sectors, market-based mechanisms, and social organizations to build a protection framework with clearly delineated responsibilities and well-coordinated roles. As the foundational platform led by the state, the BMI fulfills its universal protection function, prioritizing the inclusion of essential treatments for patients with rare diseases in the drug reimbursement list and policy design, thereby alleviating their baseline financial burdens.

Commercial health insurance, as a market-based supplement to the formal system, should leverage its advantages in flexible product design and accurate risk pricing. It can offer tailored, high-limit, and differentiated insurance products specifically designed to mitigate the financial risks associated with treatments for rare diseases [31,78]. Moreover, public–private partnerships, such as risk-sharing arrangements and reinsurance mechanisms, should be encouraged to enhance the stability and sustainability of commercial insurers’ participation in protecting individuals with rare diseases.

In addition, social charity organizations and policy-driven special assistance funds serve as the “third pillar” of the system. These actors can provide a safety net and complementary support in areas that formal institutional coverage cannot adequately reach. By establishing a multi-stakeholder resource coordination platform that integrates charitable donations, medical assistance, and clinical funds, the system can expand its coverage scope and improve payment flexibility.

In summary, the protection framework for patients with existing rare diseases should adopt a triadic collaborative model driven by government leadership, market regulation, and societal support. This structure enables vertical stratification of the system (i.e., basic, supplementary, and safety net coverage) while also fostering horizontal integration of resources and risk-sharing mechanisms. The result is a more resilient, adaptable, and equitable system, providing patients with rare diseases a stable and sustainable support framework.

### 4.5. Limitations and Future Research Directions

Despite the strong theoretical foundation and policy framework presented in this study for the DISRD, its operational effectiveness and long-term sustainability require continuous testing and refinement through policy practice and future research.

First, in terms of institutional modeling, this study has developed a comprehensive framework encompassing financing structures, responsibility sharing, benefit mechanisms, and payment pathways, introducing institutional tools such as tiered funding mechanisms and risk retention designs. However, key parameters—such as disease and drug inclusion standards, premium calculation methods, and risk-sharing structures—have not yet been systematically modeled or empirically simulated, nor have they been integrated with region-specific epidemiological and fiscal data for actuarial analysis. Future research could leverage pilot-generated real-world data to conduct scenario simulations, optimize actuarial models, and refine benefit structures, thereby enhancing fiscal sustainability, benefit equity, and policy responsiveness.

Second, while the voluntary enrollment mechanism enhances policy acceptance and flexibility, it may face challenges in constructing the risk pool. However, this study proposes multiple strategies, including “continuous enrollment incentives,” “multi-cycle renewal options” (e.g., one-year, three-year, five-year plans), and “low-income subsidy mechanisms” to mitigate this issue. The effectiveness of these strategies still needs to be observed and evaluated in practice, with further validation through behavioral data to assess the sustainability of the incentive mechanisms.

Third, in terms of moral hazard control, patients may alter their behavior due to misunderstandings of insurance coverage, leading to resource waste or unnecessary medical services. This study aims to mitigate such issues by incorporating “risk retention” mechanisms, such as deductibles, co-payment ratios, and screening cost caps, which strike a balance between service accessibility and limiting unnecessary resource use. However, future research should focus on systematically modeling the relationship between parameter settings, patient behaviors, and resource efficiency, and developing differentiated risk control mechanisms based on population, disease types, and drug characteristics to achieve a more precise balance between comprehensive coverage and resource efficiency.

Fourth, the sustained advancement of the DISRD depends on the deep engagement and collaborative support from multiple stakeholders. Future research could focus on optimizing payment mechanisms for pharmaceutical companies (e.g., pay-for-performance, phased payments, data-sharing incentives), enhancing the incentives for medical institutions in screening and referral processes, and linking performance assessments with resource allocation to improve execution capacity among health insurance, health, and civil affairs departments. Public education and policy outreach should also be strengthened, alongside systematic evaluations of the roles of patient organizations in policy communication, ethical oversight, and public participation. This would help reinforce the social foundation and moral legitimacy of the DISRD. Additionally, future research may explore governance models that institutionalize multi-stakeholder collaboration in rare disease policy implementation.

Grounded in China’s institutional context, this research proposes a pragmatic model for protecting individuals with rare diseases. Its conceptual innovations and policy design offer valuable reference points for other countries seeking to establish resilient, prevention-oriented public health systems that can effectively manage rare and high-cost conditions from an early intervention perspective.

## Figures and Tables

**Table 1:** Examples of high-cost rare disease drugs not covered by the National Reimbursement Drug List (NRDL).

Rare Disease Name	Drug Name	Annual Cost (10,000 CNY)	Approval Date
Arginase Deficiency	Glycerol Phenylbutyrate Oral Liquid	44	2023
	Sodium Phenylbutyrate Granules		2021
Citrullinemia	Sodium Phenylbutyrate Granules	44	2021
	Glycerol Phenylbutyrate Oral Liquid		2023
Familial Mediterranean Fever	Anakinra injection	21.84	2023
Gaucher’s Disease	Imiglucerase for Injection	87	2008
	Velaglucerase Alfa for Injection		2021
Glycogen Storage Disease (Type II)	Alglucosidase Alfa for Injection	127	2015
	Avalglucosidase Alfa for Injection		2023
Hyperornithinaemia--Hyperammonaemia Homocitrullinuria Syndrome	Glycerol Phenylbutyrate Oral Liquid	44	2023
	Sodium Phenylbutyrate Granules		2021
Isovaleric Acidemia	Carglumic Acid Dispersible Tablets	35.478	2023
Methylmalonic Academia			
N-acetylglutamate Synthase Deficiency			
Propionic Acidemia			
Mucopolysaccharidosis	Laronidase concentrated solution for infusion	197	2020
	Idursulfase beta Injection	97	2020
Progressive Muscular Dystrophy	Vamorolone Oral Suspension	30	2024
Ornithine Transcarbamylase Deficiency	Glycerol Phenylbutyrate Oral Liquid	44	2023
	Sodium Phenylbutyrate Granules		2021
Hyperphenylalaninemia	Sapropterin Dihydrochloride Tablets	11	2023
Phenylketonuria			
Tetrahydrobiopterin Deficiency			
Alagille syndrome	Maralixibat Chloride Oral Solution	22.5	2023
Neuroblastoma	Naxitamab Injection	60–160	2022
	Dinutuximab beta Injection	60–149	2021
Neurotrophic keratitis	Cenegermin eye drops	14	2020
Tumor-induced osteomalacia	Burosumab Injection	155	2021

Note: Data compiled by the author based on the 2025 China Rare Disease Trend Report. Annual treatment costs were estimated based on standard adult dosing.

**Table 2:** Key national policies on rare disease healthcare protection in China (2012–2025).

Policy Title	Year	Thematic Categories	Related Content
Notice on the Implementation of the New Rural Cooperative Medical Scheme (NRCMS) in 2012 [18]	2012	Institutional structure; Payment mechanisms	Twelve diseases, including hemophilia, were prioritized in a primary disease protection pilot, with reimbursement reaching about 70% of provincial cost limits. The complementary roles of NRCMS, medical assistance, commercial health insurance, and social charity should be fully leveraged to establish a stable mechanism for protecting against major and severe diseases.
Notice on the Guidelines for Improving the Medical Assistance System and Comprehensive Implementation of Catastrophic Disease Medical Aid [19]	2015	Institutional structure	Introduced financial aid for households facing severe economic hardship due to excessive medical expenses.
Notice on the Release of the First List of Rare Diseases [20]	2018	Inclusion of diseases and drugs	Officially recognized 121 rare diseases, establishing a foundational framework for policy interventions.
Work Plan for the 2019 Adjustment of the NRDL [21]	2019	Inclusion of diseases and drugs	Explicitly prioritized the inclusion of therapies for cancer, rare diseases, and other severe conditions in the NRDL.
Opinions on Deepening the Reform of the Medical Security System [22]	2020	Institutional structure	Set the goal of establishing a multi-tier medical security system by 2030, including basic medical insurance (BMI), commercial health insurance, medical assistance, and supplementary schemes, with integrated mechanisms to improve access to orphan drugs.
Opinions on Implementing a Standardized Healthcare Benefits Catalog [23]	2021	Payment mechanisms; Institutional structure	Classified certain rare diseases under special outpatient categories or chronic disease management for outpatient reimbursement, with supplementary mechanisms such as medical assistance playing a full role in covering gaps not covered by BMI.
Opinions on Improving the Medical Insurance and Assistance System for Catastrophic Diseases [24]	2021	Institutional structure;	Proposed a triple-layer protection framework—BMI, critical illness insurance, and medical assistance—while encouraging integration with charitable and commercial insurance for rare disease patients.
Work Plan for Adjusting the NRDL(Annual Iterations) [25,26,27]	2022–2024	Inclusion of diseases and drugs; Intervention timing	Specified that drugs approved by the National Medical Products Administration (NMPA) for rare disease treatment could be considered for inclusion in the reimbursement list.
Notice on the Release of the Second List of Rare Diseases [28]	2023	Inclusion of diseases and drugs	Expanded the list to include 86 additional rare diseases.
Guiding Opinions on Strengthening the Effective Integration of Government Assistance and Charitable Support [29]	2023	Institutional structure	Strengthening the integration of government assistance with charitable resources to provide precise medical coverage and charitable aid to patients with severe and rare diseases.
Notice on the Issuance of the 2024 Central Government Financial Support Plan for Social Organizations’ Participation in Social Services Projects [30]	2024	Institutional structure	The central government provides financial support for social organizations participating in rare disease medical guarantee projects, providing medical services, rehabilitation support, and treatment assistance through these organizations.
Guiding Opinions on Promoting the High-Quality Development of Inclusive Insurance [31]	2024	Institutional structure	Increasing insurance coverage for high-risk groups such as patients with rare diseases, women with “two cancers,” and children with congenital diseases, and encouraging insurance companies to offer customized medical insurance products.
Notice on the Release of the NRDL [32]	2024	Payment mechanisms	Inclusion of 126 rare disease therapies along with their reimbursement standards.

**Table 3:** International institutional arrangements for rare disease coverage.

Country/Region	Basic Coverage Mechanism	Targeted Supplementary Mechanism	Financing of the Supplementary Mechanism
Germany [47]	Statutory Health Insurance (SHI)	-	-
Turkey [48]	General Health Insurance (GHI)	-	-
United States [14]	Medicare and Medicaid	Private Health Insurance	Private insurance premiums
United Kingdom [49]	National Health Service (NHS)	CDF	Department of Health and Social Care earmarked budget
Belgium [10]	Compulsory Health Insurance	Special Solidarity Fund	Compulsory health insurance
Australia [11]	Medicare (covers basic services and PBS-listed drugs)	LSDP	Federal government appropriation
Japan [12]	National Health Insurance (NHI) and Employees’ Health Insurance(EHI)	Medical Care Program for Specific Diseases	Consumption tax
Taiwan (China) [13]	National Health Insurance (NHI)	Rare Disease Prevention and Control Fund	Tobacco tax

Note: “-” indicates that no formally documented mechanism or policy is currently in place.

## Data Availability

The original contributions presented in this study are included in the article/Supplementary Materials. Further inquiries can be directed to the corresponding author.

## Supplementary Materials

The following supporting information can be downloaded at: https://www.mdpi.com/article/10.3390/healthcare13172178/s1, Table S1: The policy source for the outpatient reimbursement ceiling of basic medical insurance.

## Abbreviations

The following abbreviations are used in this manuscript:

NRDL	National Reimbursement Drug List
BMI	Basic Medical Insurance
URRBMI	Rural Resident Basic Medical Insurance
UEBMI	Urban Employee Basic Medical Insurance
WHO	World Health Organization
MOF	Ministry of Finance
NHSA	National Healthcare Security Administration
R&D	pharmaceutical research and development
NRCMS	New Rural Cooperative Medical Scheme
CDF	Cancer Drugs Fund
LSDP	Life Saving Drugs Program
DISRD	Dedicated Insurance Scheme for Rare Disease

## Appendix A

**Table A1:** Rare disease drugs included in the NDRL.

Drug Name	Drug Category (NRDL)	Rare Disease Name
Riluzole	Category B drugs	Amyotrophic Lateral Sclerosis
Riluzole Oral Suspension	Category B drugs	Amyotrophic Lateral Sclerosis
Edaravone Injection	Category B drugs	Amyotrophic Lateral Sclerosis
Edaravone and Sodium Chloride Injection	Category B drugs	Amyotrophic Lateral Sclerosis
Eculizumab Injection	Category B drugs	Atypical Hemolytic Uremic Syndrome, Hemophilia, Paroxysmal Nocturnal Hemoglobinuria
Human Immunoglobulin (pH4) for Intravenous Injection	Category B drugs	Autoimmune Encephalitis, Gaucher’s Disease, Primary Combined Immune Deficiency, X-linked Agammaglobulinemia
Levocarnitine	Category B drugs	Carnitine Deficiency
Siltuximab for Injection	Category B drugs	Castleman Disease
Hydrocortisone	Category A drugs	21-Hydroxylase Deficiency, Congenital Adrenal Hypoplasia
Agalsidase Alfa Concentrated Solution for Infusion	Category B drugs	Fabry Disease
Eliglustat Tartrate Capsules	Category B drugs	Gaucher’s Disease
Pyridostigmine Bromide	Category A drugs	Gaucher’s Disease
Efgartigimod Alfa Injection (Subcutaneous Injection)	Category B drugs	Generalized Myasthenia Gravis
Desmopressin	Category A drugs	Hemophilia
Desmopressin Oral Solution	Category B drugs	Hemophilia
Human Coagulation Factor VIII	Category A drugs	Hemophilia
Human Prothrombin Complex	Category B drugs	Hemophilia, Congenital factor VII deficiency
Recombinant Human Coagulation Factor VIII	Category B drugs	Hemophilia
Recombinant Coagulation Factor IX	Category B drugs	Hemophilia
Human Coagulation Factor IX	Category B drugs	Hemophilia
Tranexamic Acid	Category A/B drugs	Hemophilia, Hereditary Angioedema (HAE)
Penicillamine	Category A drugs	Hepatolenticular Degeneration (Wilson Disease)
Dimercaptosuccinic Acid	Category A drugs	Hepatolenticular Degeneration (Wilson Disease)
Disulfide Butane Disodium	Category A drugs	Hepatolenticular Degeneration (Wilson Disease)
Zinc Sulfate Tablets	Category B drugs	Hepatolenticular Degeneration (Wilson Disease)
Danazol	Category B drugs	Hereditary Angioedema (HAE)
Lanadelumab Injection	Category B drugs	Hereditary Angioedema (HAE)
Icatibant Acetate Injection	Category B drugs	Hereditary Angioedema (HAE)
Potassium Aspartate and Magnesium Aspartate	Category B drugs	Hereditary Hypomagnesemia
Rosuvastatin	Category B drugs	Homozygous Hypercholesterolemia
Atorvastatin	Category B drugs	Homozygous Hypercholesterolemia
Ezetimibe	Category B drugs	Homozygous Hypercholesterolemia, Sitosterolemia
Simvastatin	Category A drugs	Homozygous Hypercholesterolemia
Evolocumab Injection	Category B drugs	Homozygous Hypercholesterolemia
Ezetimibe and Atorvastatin Calcium Tablets (II)	Category B drugs	Homozygous Hypercholesterolemia
Rosuvastatin Calcium and Ezetimibe Tablets (I)	Category B drugs	Homozygous Hypercholesterolemia
Deutetrabenazine Tablets	Category B drugs	Huntington Disease
Tetrabenazine Tablets	Category B drugs	Huntington Disease
Calcitriol	Category B drugs	Hypophosphatemic Rickets
Calcitriol Oral Solution	Category B drugs	Hypophosphatemic Rickets
Tafamidis Meglumine Soft Capsules	Category B drugs	Idiopathic Cardiomyopathy, Transthyretin amyloidosis
Tafamidis Soft Capsules	Category B drugs	Idiopathic Cardiomyopathy, Transthyretin amyloidosis
Mavacamten Capsules	Category B drugs	Idiopathic Cardiomyopathy
Gonadorelin	Category B drugs	Idiopathic Hypogonadotropic Hypogonadism, Kallmann Syndrome
Chorionic Gonadotrophin	Category A drugs	Idiopathic Hypogonadotropic Hypogonadism, Kallmann Syndrome
Menotropins	Category B drugs	Idiopathic Hypogonadotropic Hypogonadism, Kallmann Syndrome
Testosterone Undecanoate	Category B drugs	Idiopathic Hypogonadotropic Hypogonadism, Kallmann Syndrome
Bosentan Dispersible Tablets	Category B drugs	Idiopathic Pulmonary Arterial Hypertension
Bosentan Tablets	Category B drugs	Idiopathic Pulmonary Arterial Hypertension
Riociguat Tablets	Category B drugs	Idiopathic Pulmonary Arterial Hypertension
Macitentan Tablets	Category B drugs	Idiopathic Pulmonary Arterial Hypertension
Selexipag Tablets	Category B drugs	Idiopathic Pulmonary Arterial Hypertension
Ambrisentan	Category B drugs	Idiopathic Pulmonary Arterial Hypertension
Treprostinil Injection	Category B drugs	Idiopathic Pulmonary Arterial Hypertension
Beraprost Sodium Sustained-release Tablets	Category B drugs	Idiopathic Pulmonary Arterial Hypertension
Nintedanib Esilate Soft Capsules	Category B drugs	Idiopathic Pulmonary Fibrosis, Systemic Sclerosis, Progressive fibrosing interstitial lung disease
Pirfenidone	Category B drugs	Idiopathic Pulmonary Fibrosis
Teriflunomide Tablets	Category B drugs	Multiple Sclerosis
Siponimod Tablets	Category B drugs	Multiple Sclerosis
Fingolimod Hydrochloride Capsules	Category B drugs	Multiple Sclerosis
Fampridine Sustained-Release Tablets	Category B drugs	Multiple Sclerosis
Dimethyl Fumarate Enteric Capsules	Category B drugs	Multiple Sclerosis
Ofatumumab Injection	Category B drugs	Multiple Sclerosis
Ozanimod Hydrochloride Capsules	Category B drugs	Multiple Sclerosis
Insulin	Category A/B drugs	Neonatal Diabetes Mellitus
Glibenclamide	Category A drugs	Neonatal Diabetes Mellitus
Satralizumab Injection	Category B drugs	Neuromyelitis Optica
Inebilizumab Injection	Category B drugs	Neuromyelitis Optica
Miglustat Capsules	Category B drugs	Niemann-Pick Disease
Levodopa	Category A drugs	Parkinson Disease (Young-onset, Early-onset)
Amantadine	Category A drugs	Parkinson Disease (Young-onset, Early-onset)
Levodopa and Benserazide Hydrochloride	Category A drugs	Parkinson Disease (Young-onset, Early-onset)
Carbidopa and Levodopa	Category B drugs	Parkinson Disease (Young-onset, Early-onset)
Carbidopa	Category B drugs	Parkinson Disease (Young-onset, Early-onset)
Droxidopa	Category B drugs	Parkinson Disease (Young-onset, Early-onset)
Ropinirole	Category B drugs	Parkinson Disease (Young-onset, Early-onset)
Pramipexole	Category B drugs	Parkinson Disease (Young-onset, Early-onset)
Selegiline	Category B drugs	Parkinson Disease (Young-onset, Early-onset)
Rasagiline	Category B drugs	Parkinson Disease (Young-onset, Early-onset)
Entacapone, Levodopa and Carbidopa Tablets	Category B drugs	Parkinson Disease (Young-onset, Early-onset)
Trihexyphenidyl Hydrochloride	Category A drugs	Parkinson Disease (Young-onset, Early-onset)
Iptacopan Hydrochloride Capsules	Category B drugs	Paroxysmal Nocturnal Hemoglobinuria
Daratumumab Injection (Subcutaneous Injection)	Category B drugs	Primary Light Chain Amyloidosis
Ursodeoxycholic Acid	Category A drugs	Progressive Familial Intrahepatic Cholestasis, Primary biliary cholangitis, Primary sclerosing cholangitis
Mecapegfilgrastim Injection	Category B drugs	Severe Congenital Neutropenia
Efbemalenograstim alfa Injection	Category B drugs	Severe Congenital Neutropenia
Telpegfilgrastim Injection	Category B drugs	Severe Congenital Neutropenia
Stiripentol for Suspension	Category B drugs	Severe Myoclonic Epilepsy in Infancy (Dravet Syndrome)
Nusinersen Sodium Injection	Category B drugs	Spinal Muscular Atrophy
Risdiplam Powder for Oral Solution	Category B drugs	Spinal Muscular Atrophy
Everolimus Tablets	Category B drugs	Tuberous Sclerosis Complex
Sirolimus Gel	Category B drugs	Tuberous Sclerosis Complex
Nitisinone Capsules	Category B drugs	Tyrosinemia
Recombinant Human Coagulation Factor VIIa for Injection (Eptacog alfa, activated)	Category B drugs	Hemophilia, Congenital factor VII deficiency, Glanzmann thrombasthenia
Octreotide Acetate Microspheres for Injection	Category B drugs	Acromegaly
Lanreotide acetate sustained-release injection (a pre-filled syringe)	Category B drugs	Acromegaly, Gastroenteropancreatic neuroendocrine neoplasm
Brentuximab Vedotin for Injection	Category B drugs	Cutaneous T-cell lymphomas
Methoxsalen	Category B drugs	Cutaneous T-cell lymphomas
Imatinib	Category B drugs	Dermatofibrosarcoma protuberans
Surufatinib Capsules	Category B drugs	Gastroenteropancreatic neuroendocrine neoplasm, Multiple endocrine neoplasia
Sunitinib	Category B drugs	Gastrointestinal stromal tumor
Ripretinib Tablets	Category B drugs	Gastrointestinal stromal tumor
Avapritinib Tablets	Category B drugs	Gastrointestinal stromal tumor
spesolimab injection	Category B drugs	Generalized pustular psoriasis
Narlumosbart Injection	Category B drugs	Giant cell tumor of bone
Denosumab Injection	Category B drugs	Giant cell tumor of bone
Temozolomide Capsules	Category B drugs	Glioblastoma
Vebreltinib Enteric Capsules	Category B drugs	Glioblastoma
Clobazam Tablets	Category B drugs	Lennox-Gastaut syndrome
Lamotrigine	Category B drugs	Lennox-Gastaut syndrome
Pemetrexed	Category B drugs	Malignant pleural mesothelioma
Dabrafenib mesylate Capsules	Category B drugs	Melanoma
Trametinib tablets	Category B drugs	Melanoma
Vemurafenib film-coated tablets	Category B drugs	Melanoma
Tunlametinib Capsules	Category B drugs	Melanoma
Pitolisant Hydrochloride Tablets	Category B drugs	Narcolepsy
Selumetinib Hydrogen Sulfate Capsules	Category B drugs	Neurofibromatosis
Ruxolitinib Phosphate Tablets	Category B drugs	Polycythaemia vera, Primary myelofibrosis
Recombinant Human Growth Hormone	Category B drugs	Primary ciliary dyskinesia
Ranibizumab Injections	Category B drugs	Retinopathy of prematurity
Tocilizumab	Category B drugs	Systemic juvenile idiopathic arthritis
Luspatercept for Injection	Category B drugs	Thalassemia major
Deferasirox Granules	Category B drugs	Thalassemia major
Zanubrutinib Capsules	Category B drugs	Waldenström macroglobulinemia/ Lymphoplasmacytic lymphoma
Ibrutinib Capsules	Category B drugs	Waldenström macroglobulinemia/ Lymphoplasmacytic lymphoma
Vigabatrin Powder for Oral Solution	Category B drugs	West syndrome/Infantile spasms syndrome

Note: Category A drugs are fully reimbursed under China’s Basic Medical Insurance (BMI) scheme, while Category B drugs require partial out-of-pocket payment from patients.

## References

[1] Valdez R., Ouyang L., Bolen J. (2016). Public Health and Rare Diseases: Oxymoron No More. Prev. Chronic Dis..

[2] Wang X., Li S.-C., Yue X., Li Y., Shi N., Zhao F.-L., Wu J. (2023). Patient Access to Orphan Drugs Covered by Medical Insurance in China: Real-World Evidence from Patient Survey. Value Health Reg. Issues.

[3] Cai X., Yang H., Genchev G.Z., Lu H., Yu G. (2019). Analysis of Economic Burden and Its Associated Factors of Twenty-Three Rare Diseases in Shanghai. Orphanet J. Rare Dis..

[4] Nguengang Wakap S., Lambert D.M., Olry A., Rodwell C., Gueydan C., Lanneau V., Murphy D., Le Cam Y., Rath A. (2020). Estimating Cumulative Point Prevalence of Rare Diseases: Analysis of the Orphanet Database. Eur. J. Hum. Genet..

[5] Guo J., Liu P., Chen L., Lv H., Li J., Gu W., Xu K., Zhu Y., Wu Z., Tian Z. (2021). National Rare Diseases Registry System (NRDRS): China’s First Nation-Wide Rare Diseases Demographic Analyses. Orphanet J. Rare Dis..

[6] Qiao L., Liu X., Shang J., Zuo W., Xu T., Qu J., Jiang J., Zhang B., Zhang S. (2022). Evaluating the National System for Rare Diseases in China from the Point of Drug Access: Progress and Challenges. Orphanet J. Rare Dis..

[7] Zhao Z., Pei Z., Hu A., Zhang Y., Chen J. (2023). Analysis of Incentive Policies and Initiatives on Orphan Drug Development in China: Challenges, Reforms and Implications. Orphanet J. Rare Dis..

[8] Xu K., Evans D.B., Kawabata K., Zeramdini R., Klavus J., Murray C.J.L. (2003). Household Catastrophic Health Expenditure: A Multicountry Analysis. Lancet.

[9] Li C., Young B.-R., Jian W. (2018). Association of Socioeconomic Status with Financial Burden of Disease among Elderly Patients with Cardiovascular Disease: Evidence from the China Health and Retirement Longitudinal Survey. BMJ Open.

[10] Ng Q.X., Ong C., Chan K.E., Ong T.S.K., Lim I.J.X., Tang A.S.P., Chan H.W., Koh G.C.H. (2024). Comparative Policy Analysis of National Rare Disease Funding Policies in Australia, Singapore, South Korea, the United Kingdom, and the United States: A Scoping Review. Health Econ. Rev..

[11] Department of Health and Aged Care About the Life Saving Drugs Programme. https://www.health.gov.au/our-work/life-saving-drugs-program/about-the-lsdp.

[12] Nakada H., Watanabe S., Takashima K., Suzuki S., Kawamura Y., Takai Y., Matsui K., Yamamoto K. (2023). General Public’s Understanding of Rare Diseases and Their Opinions on Medical Resource Allocation in Japan: A Cross-Sectional Study. Orphanet J. Rare Dis..

[13] Lin T.-F., Chan Y., Chen J. (2020). Awareness of Tobacco Tax Policy and Public Opinion on Tobacco Tax Reform in Taiwan. Asian Pac. J. Health Econ. Policy.

[14] Thomas S., Caplan A. (2019). The Orphan Drug Act Revisited. JAMA.

[15] Dharssi S., Wong-Rieger D., Harold M., Terry S. (2017). Review of 11 National Policies for Rare Diseases in the Context of Key Patient Needs. Orphanet J. Rare Dis..

[16] Williams C.A., Heins R.M. (1985). Risk Management and Insurance.

[17] Grossman M. (1972). On the Concept of Health Capital and the Demand for Health. J. Polit. Econ..

[18] Ministry of Finance of China Notice on the Implementation of the New Rural Cooperative Medical Scheme in 2012. https://www.mof.gov.cn/gkml/bulinggonggao/tongzhitonggao/201205/t20120528_654516.htm.

[19] The General Office of the State Council Notice on the Guidelines for Improving the Medical Assistance System and Comprehensive Implementation of Catastrophic Disease Medical Aid. https://www.gov.cn/gongbao/content/2015/content_2864053.htm.

[20] National Health Commission of the People’s Republic of China Notice on the Release of the First List of Rare Diseases. https://www.gov.cn/zhengce/zhengceku/2018-12/31/content_5435167.htm.

[21] National Healthcare Security Administration Work Plan for the 2019 Adjustment of the National Reimbursement Drug List. https://www.gov.cn/zhengce/zhengceku/2019-04/17/content_5562265.htm.

[22] The State Council of the People’s Republic of China Opinions on Deepening the Reform of the Medical Security System. https://www.gov.cn/gongbao/content/2020/content_5496762.htm.

[23] National Healthcare Security Administration, Ministry of Finance of China Opinions on Implementing a Standardized Healthcare Benefits Catalog. https://www.gov.cn/zhengce/zhengceku/2021-08/11/content_5630791.htm.

[24] The General Office of the State Council Opinions on Improving the Medical Insurance and Assistance System for Major and Severe Diseases. https://www.gov.cn/zhengce/zhengceku/2021-11/19/content_5651446.htm.

[25] National Healthcare Security Administration Work Plan for Adjusting the National Reimbursement Drug List in 2022. https://www.gov.cn/zhengce/zhengceku/2022-06/30/content_5698559.htm.

[26] National Healthcare Security Administration Work Plan for Adjusting the National Reimbursement Drug List in 2023. https://www.gov.cn/zhengce/zhengceku/202306/content_6889132.htm.

[27] National Healthcare Security Administration Work Plan for Adjusting the National Reimbursement Drug List in 2024. https://www.gov.cn/zhengce/zhengceku/202406/content_6960127.htm.

[28] National Health Commission of the People’s Republic of China Notice on the Release of the Second List of Rare Diseases. https://www.gov.cn/zhengce/zhengceku/202309/content_6905273.htm.

[29] Ministry of Civil Affairs of the People’s Republic of China Guiding Opinions on Strengthening the Effective Integration of Government Assistance and Charitable Support. https://www.gov.cn/zhengce/zhengceku/202309/content_6902442.htm.

[30] Ministry of Civil Affairs of the People’s Republic of China Notice on the Issuance of the 2024 Central Government Financial Support Plan for Social Organizations’ Participation in Social Services Projects. https://www.mca.gov.cn/gdnps/pc/content.jsp?id=1662004999979999113.

[31] The State Council of the People’s Republic of China Guiding Opinions on Promoting the High-Quality Development of Inclusive Insurance. https://www.gov.cn/zhengce/zhengceku/202406/content_6955980.htm.

[32] National Healthcare Security Administration, Ministry of Human Resources and Social Security (2024). Notice on the Release of the National Reimbursement Drug List. https://www.gov.cn/zhengce/zhengceku/202411/content_6989859.htm.

[33] Li J., Yang L., Zhang Y., Liao H., Ma Y., Sun Q. (2022). Rare Disease Curative Care Expenditure-Financing Scheme-Health Provider–Beneficiary Group Analysis: An Empirical Study in Sichuan Province, China. Orphanet J. Rare Dis..

[34] Ma L., Chen Y., Ding J. (2024). The current situation, problems, and optimization countermeasures of the basic medical insurance treatment of orphan drugs in China. World Clin. Drug.

[35] Xu J., Yu M., Zhang Z., Gong S., Li B. (2023). Is Sub-National Healthcare Social Protection Sufficient for Protecting Rare Disease Patients? The Case of China. Front. Public Health.

[36] Sullivan Alliance for Pain Relief Observation Report on the Trends of China’s Rare Diseases. https://www.frostchina.com/content/insight/detail/67bed1ec7ed30cc08c184b97.

[37] Chen Y., Chen X., Deng Y., Ding J. (2024). Analysis of Affordability Differences for Rare Diseases in China: A Comparison across Disease Types and Regions. Int. J. Equity Health.

[38] Melmeyer P. (2016). Adverse Selection in Health Insurance Coverage of High-Cost Cures. Policy Perspect..

[39] Wu J., Qiao J., Nicholas S., Liu Y., Maitland E. (2022). The Challenge of Healthcare Big Data to China’s Commercial Health Insurance Industry: Evaluation and Recommendations. BMC Health Serv. Res..

[40] Gao F., Powers M.R., Wang J. (2009). Adverse Selection or Advantageous Selection? Risk and Underwriting in China’s Health-Insurance Market. Insur. Math. Econ..

[41] Liu M., Lu Y., Li J., Zhang Y., Zhang S., Liu Y. (2024). Orphan Drug Policy Analysis in China. Front. Pharmacol..

[42] Ma Z., Wen X. (2025). The Positioning, Value, Development Trend, Problems and Suggestions of Commercial Health Insurance in China Under the Healthy China Initiative. China Med. Insur..

[43] Yan X., He S., Dong D. (2020). Determining How Far an Adult Rare Disease Patient Needs to Travel. for a Definitive Diagnosis: A Cross-Sectional Examination of the 2018 National Rare Disease Survey in China. Int. J. Environ. Res. Public Health.

[44] Zhang S., Dong D. (2020). 2020 China Rare Diseases Comprehensive Social Survey.

[45] Dumbuya J.S., Chen X., Deng L., Ahmad B., Lu J. (2025). A Retrospective Study of Insurance Coverage Status and Economic Cost of Rare Diseases in Hainan Province. Sci. Rep..

[46] Chen Y., Chen X., Deng Y., Yan J., Li J., Ding J. (2024). HPR65 Assessing Rare Disease Reimbursement Levels and Economic Burden on Patients in China. Value Health.

[47] Bock J.-O., Matschinger H., Brenner H., Wild B., Haefeli W.E., Quinzler R., Saum K.-U., Heider D., König H.-H. (2014). Inequalities in Out-of-Pocket Payments for Health Care Services among Elderly Germans—Results of a Population-Based Cross-Sectional Study. Int. J. Equity Health.

[48] Koçkaya G., Wertheimer A.I., Kilic P., Tanyeri P., Vural I.M., Akbulat A., Artiran G., Kerman S. (2014). An Overview of the Orphan Medicines Market in Turkey. Value Health Reg. Issues.

[49] He J., Zhang Y., Xia S., Hu S. (2012). Social security system of rare diseases in European Union and its implications for China. Chin. J. Health Policy.

[50] Nord E., Richardson J., Street A., Kuhse H., Singer P. (1995). Who Cares about Cost? Does Economic Analysis Impose or Reflect Social Values?. Health Policy.

[51] Li X., Lu Z., Zhang J., Zhang X., Zhang S., Zhou J., Li B., Ou L. (2020). The Urgent Need to Empower Rare Disease Organizations in China: An Interview-Based Study. Orphanet J. Rare Dis..

[52] Li X., Zhang X., Zhang S., Lu Z., Zhang J., Zhou J., Li B., Ou L. (2021). Rare Disease Awareness and Perspectives of Physicians in China: A Questionnaire-Based Study. Orphanet J. Rare Dis..

[53] R&D-Based Pharmaceutical Association Committee (RDPAC) of the China Association of Enterprises with Foreign Investment Research Report on the Experience and Mechanism of Diversified Protection Policies for Rare Diseases in My Country. https://cnadmin.rdpac.org/upload/upload_file/1677491593.pdf.

[54] National Healthcare Security Administration Notice on Advancing Basic Medical Insurance for Urban and Rural Residents in 2024. https://www.gov.cn/zhengce/zhengceku/202408/content_6970593.htm.

[55] The General Office of the State Council Guiding Opinions on Improving the Sustainable Participation Mechanism in Basic Medical Insurance. https://www.gov.cn/gongbao/2024/issue_11526/202408/content_6969189.html.

[56] He J., Song P., Kang Q., Zhang X., Hu J., Yang Y., Tang M., Chen D., Hu S., Jin C. (2019). Overview on Social Security System of Rare Diseases in China. Biosci. Trends.

[57] Kanatani Y., Tomita N., Sato Y., Eto A., Omoe H., Mizushima H. (2017). National Registry of Designated Intractable Diseases in Japan: Present Status and Future Prospects. Neurol. Med. Chir..

[58] The State Council of the People’s Republic of China The Outline of the Healthy China 2030 National Plan. https://www.gov.cn/zhengce/202203/content_3635233.htm.

[59] National Health Commission of the People’s Republic of China Issuing the Birth Defects Prevention and Control Capacity Building Plan (2023–2027). https://www.gov.cn/zhengce/zhengceku/202308/content_6900320.htm.

[60] Igor F. (2023). Selection on Moral Hazard in the Swiss Market for Mandatory Health Insurance: Empirical Evidence from Swiss Household Panel Data. arXiv.

[61] Wain K., Ritzwoller D.P., Perraillon M.C. (2025). Quantifying Changes to Healthcare Utilization after a Reduction in Cost-Sharing among Deductible Plan Enrollees. arXiv.

[62] Eichler H.-G., Trusheim M., Schwarzer-Daum B., Larholt K., Zeitlinger M., Brunninger M., Sherman M., Strutton D., Hirsch G. (2022). Precision Reimbursement for Precision Medicine: Using Real-World Evidence to Evolve from Trial-and-Project to Track-and-Pay to Learn-and-Predict. Clin. Pharmacol. Ther..

[63] Liu J., Barrett J.S., Leonardi E.T., Lee L., Roychoudhury S., Chen Y., Trifillis P. (2022). Natural History and Real-World Data in Rare Diseases: Applications, Limitations, and Future Perspectives. J. Clin. Pharmacol..

[64] Dhiab L.B., Louail B., Allouche M.A., Abdennouz A. (2024). Analyzing the Impact of Service Quality, Technology, and Policy Management on Insurance Policy Renewal. J. Manag. World.

[65] Panda P., Chakraborty A., Raza W., Bedi A.S. (2016). Renewing Membership in Three Community-Based Health Insurance Schemes in Rural India. Health Policy Plan..

[66] Hussien M., Azage M. (2021). Barriers and Facilitators of Community-Based Health Insurance Policy Renewal in Low- and Middle-Income Countries: A Systematic Review. Clin. Outcomes Res..

[67] Rahim K.N.K.A., Lattepi N.M., Sarimin R., Shir F.S., Akmal S., Wai L.S., Ghazali I.M.M. (2024). Development of an Multicriteria Decision Analysis Framework for Rare Disease Reimbursement Prioritization in Malaysia. Int. J. Technol. Assess. Health Care.

[68] Chen H., Xiang Y., Tang X., Hu M. (2024). Establishment of a Value Assessment Framework for Orphan Medicinal Products in China. Orphanet J. Rare Dis..

[69] Nicod E., Kanavos P. (2016). Scientific and Social Value Judgments for Orphan Drugs in Health Technology Assessment. Int. J. Technol. Assess. Health Care.

[70] Blonda A., Denier Y., Huys I., Simoens S. (2021). How to Value Orphan Drugs? A Review of European Value Assessment Frameworks. Front. Pharmacol..

[71] Iskrov G., Miteva-Katrandzhieva T., Stefanov R. (2016). Multi-Criteria Decision Analysis for Assessment and Appraisal of Orphan Drugs. Front. Public. Health.

[72] Wagner M., Khoury H., Willet J., Rindress D., Goetghebeur M. (2016). Can the EVIDEM Framework Tackle Issues Raised by Evaluating Treatments for Rare Diseases: Analysis of Issues and Policies, and Context-Specific Adaptation. Pharmacoeconomics.

[73] Schey C., Krabbe P.F.M., Postma M.J., Connolly M.P. (2017). Multi-Criteria Decision Analysis (MCDA): Testing a Proposed MCDA Framework for Orphan Drugs. Orphanet J. Rare Dis..

[74] Jakab I., Németh B., Elezbawy B., Karadayı M.A., Tozan H., Aydın S., Shen J., Kaló Z. (2020). Potential Criteria for Frameworks to Support the Evaluation of Innovative Medicines in Upper Middle-Income Countries—A Systematic Literature Review on Value Frameworks and Multi-Criteria Decision Analyses. Front. Pharmacol..

[75] Simoens S. (2014). Health Technologies for Rare Diseases: Does Conventional HTA Still Apply?. Expert. Rev. Pharmacoecon Outcomes Res..

[76] Kanters T.A., Hakkaart L., Rutten-van Mölken M.P., Redekop W.K. (2015). Access to Orphan Drugs in Western Europe: Can More Systematic Policymaking Really Help to Avoid Different Decisions about the Same Drug?. Expert. Rev. Pharmacoecon Outcomes Res..

[77] National Healthcare Security Administration Issuing the Measures to Support the High-Quality Development of Innovative Drugs. https://www.gov.cn/zhengce/zhengceku/202507/content_7030260.htm.

[78] The General Office of the State Council Opinions on Accelerating the Development of Commercial Health Insurance. https://www.gov.cn/gongbao/content/2014/content_2781470.htm.

